# Mistletoe-Based Drugs Work in Synergy with Radio-Chemotherapy in the Treatment of Glioma* In Vitro* and* In Vivo* in Glioblastoma Bearing Mice

**DOI:** 10.1155/2019/1376140

**Published:** 2019-07-03

**Authors:** Sonja Schötterl, Jennifer T. Miemietz, Elena I. Ilina, Naita M. Wirsik, Ingrid Ehrlich, Andrea Gall, Stephan M. Huber, Hans Lentzen, Michel Mittelbronn, Ulrike Naumann

**Affiliations:** ^1^Molecular Neurooncology, Hertie Institute for Clinical Brain Research and Center Neurology, University of Tübingen, Otfried-Müller-Str. 27, 72076 Tübingen, Germany; ^2^Luxembourg Centre of Neuropathology (LCNP), 1 Rue Louis Rech, L-3555 Dudelange, Luxembourg; ^3^NORLUX Neurooncology Laboratory, Luxembourg Institute of Health, 84 Rue Val Fleuri, L-1526 Luxembourg, Luxembourg; ^4^Neurological Institute (Edinger Institute), Goethe University Frankfurt, Heinrich-Hoffmann-Str. 7, 60528 Frankfurt/Main, Germany; ^5^Learning and Memory, Hertie Institute for Clinical Brain Research and Center Neurology, University of Tübingen, Otfried-Müller-Str. 27, 72076 Tübingen, Germany; ^6^Department of Radiation Oncology, University of Tübingen, Hoppe-Seyler-Str. 3, 72076 Tübingen, Germany; ^7^MELEMA Pharma GmbH, Hamburg, Germany; ^8^Laboratoire Nationale de Santé, 1 Rue Louis Rech, L-3555 Dudelange, Luxembourg; ^9^Luxembourg Centre for Systems Biomedicine (LCSB), University of Luxembourg, 7 Avenue des Hauts-Fourneaux, 4362 Esch-sur-Alzette, Luxembourg

## Abstract

**Background:**

Extracts from* Viscum album L.* (VE) are used in the complementary cancer therapy in Europe for decades. VE contain several compounds like the mistletoe lectins (MLs) 1-3 and viscotoxins and also several minor ingredients. Since mistletoe lectin 1 (ML-1) has been described as the main component of VE harboring antitumor activity, purified native or recombinant ML-1 has been recently used in clinical trials. MLs stimulate the immune system, induce cytotoxicity, are able to modify the expression of cancer-associated genes, and influence the proliferation and motility of tumor cells.

**Objective:**

In this study our goal was to determine anticancer effects of the VE ISCADOR Qu, of recombinant ML-1 (Aviscumine), and of native ML-1 in the treatment of glioblastoma (GBM), the most common and highly malignant brain tumor in adults. Additionally we were interested whether these drugs, used in combination with a temozolomide-(TMZ)-based radio-chemotherapy, provide synergistic effects.

**Methods:**

Cell culture assays,* ex vivo* murine hippocampal brain slice cultures, human GBM cryosections, and a xenograft orthotopic glioblastoma mouse model were used.

**Results:**

In cells, the expression of the ML receptor CD75s, which is also expressed in GBM specimen, but not in normal brain, correlates with the drug-induced cytotoxicity. In GBM cells, the drugs induce cell death in a concentration-dependent manner and reduce cell growth by inducing cell cycle arrest in the G_2_/M phase. The cell cycle arrest was paralleled by modifications in the expression of cell cycle regulating genes. ML containing drugs, if combined with glioma standard therapy, provide synergistic and additive anticancer effects. Despite not reaching statistical significance, a single intratumoral application of Aviscumine prolonged the median survival of GBM mice longer than tumor irradiation. Moreover, intratumorally applied Aviscumine prolonged the survival of GBM-bearing mice if used in combination with irradiation and TMZ for further 6.5 days compared to the radio-chemotherapy.

**Conclusion:**

Our results suggest that an adjuvant treatment of glioma patients with ML-containing drugs might be beneficial.

## 1. Introduction

Glioblastoma (GBM) is the most common malignant WHO grade IV brain tumour with an infaust prognosis. The current standard therapy includes tumour resection, followed by irradiation and chemotherapy, using the DNA alkylating agent TMZ. However, the median survival time, even at optimal surgical resection of the tumour and at optimal conditions, is less than 20 months [1]. Novel therapy approaches targeting tumor neoangiogenesis, immune surveillance, or GBM invasion are in progress. However, until today no outstanding effects on the survival of GBM patients have been achieved by novel therapies. The failure of several new therapy approaches is mainly based on GBM characteristics like its diffuse, infiltrative growth into the brain parenchyma, its strong proliferation, massive immunosuppression, high angiogenic capacity, and its multi-drug-resistance, at least in recurrent glioma and glioma stem cells [2]. In this context, the development of drugs or identification of (natural) compounds that work in synergy with glioma standard therapy or even with novel therapeutic approaches is necessary to design an optimal therapeutic regimen for GBM patients.

Aqueous VE are used as adjuvant cancer treatment agents for decades, especially in European countries. The contents of these extracts vary dependent on the brand (e.g., ISCADOR, AbnobaVISCUM, and Helixor) due to differences in the manufacturing process. Besides, the host tree and season in which the plant is harvested also influence the composition. In the past, anticancer effects of VE were supposed to be mainly mediated by ML 1-3, being the main anticancer active component [3]. In addition to ML, viscotoxins (VT), triterpenes, flavonoids, phytosterols, and oligo- and polysaccharides are described as components of VE harbouring antitumour activity or potentiating the anticancer activity of MLs [4–8]. These minor components are not as well described as the MLs, but their effects might be still of great importance. Nevertheless one should keep in mind that some of the above-mentioned minor compounds are insoluble in water and are therefore absent or present in only very small concentrations in the standardly used aqueous extracts. In this regard also lipophilic VE were tested* in vitro* and provided promising results [9–11]. VE have been tested as an adjuvant cancer therapeutic not only* in vitro* or in tumor-bearing mice [12–18], but also in several clinical trials [19–24]. However, whether mistletoe-based drugs provide anticancer effects in patients or not is controversially discussed.

MLs are ribosomal inhibitor type 2 proteins (RIP) and consist of two glycosylated protein chains [25]. The A-chain harbours the RIP function, while the B-chain binds to glycoproteins and glycolipids bearing the glycan *α*2-6 Sialo-*N*-acetyllactosamine (CD75s) on the cell surface [26], and this mediates the uptake of the protein [27, 28]. CD75s has been described to be expressed on immune cells [29–31], and upregulated CD75s expression has been shown in colorectal, gastric, hepatocellular, lung, mammary, ovarian, and pancreatic carcinoma [32–36]. We and others demonstrated that ML-containing drugs possess several anticancer functions, apart from their mere function as a RIP. MLs provide cytotoxicity in target cells, increase anticancer immune responses, reduce tumour cell motility, modify cancer-associated gene expression, and reduce tumor growth in several mouse cancer models [12, 15, 17, 37–39]. Besides, several clinical studies have demonstrated the anticancer activity of ML or VE [21–24, 40]. However, several issues concerning the mode of action of ML or VE remain still to be elucidated. Is there a link between the expression of CD75s on target cells, the uptake of ML by target cells, and its anticancer activity? How do VE or ML mediate their anticancer effects? Do they work in synergy with radio-chemotherapy? Do we have to expect toxic side effects in the healthy brain? To answer these questions in the context of glioma we used three ML-containing drugs. ISCADOR Qu is a ML-rich VE generated of* Viscum album L*. growing on oak trees [41]. Aviscumine is recombinant, nonglycosylated ML-1 produced in* E. coli*, and native ML-1 was isolated from mistletoe plants growing on ash trees [42, 43]. In the present study we demonstrate that these drugs, even to a different extent, induce cell death and reduce glioma cell proliferation* in vitro*, work in synergy with radio-chemotherapy, and provide low toxicity in the healthy brain tissue. Furthermore, we show that CD75s expression on glioma cells correlates with their sensitivity to cell death induced by the treatment, bringing up CD75s as a putative biomarker that might predict the therapeutic efficacy of a ML-based therapy in GBM patients. Additionally, using a mouse xenograft GBM model, we show that a single intratumoral injection of Aviscumine prolonged the median survival of glioma-bearing mice, either as a single treatment option or in combination with irradiation and chemotherapy.

## 2. Materials and Methods

### 2.1. Viscumin Preparations

The ISCADOR AG (Lörrach, Germany) kindly provided ISCADOR Qu. ML and viscotoxin contents were as follows: ISCADOR Qu_20_ (Charge 4080/3: 20 mg/ml of extract, i.e., ML 1095 ng/ml, VT 48 *µ*g/ml). Aviscumine (ME-503, GMP quality), a recombinant, nonglycosylated ML-1 produced in* E. coli*, was a kind gift of H. Lentzen (MELEMA Pharma GmbH, Hamburg, Germany). Native ML-1, isolated from mistletoe plants growing on ash trees, was kindly provided by C. Heyder (Abnoba GmbH, Pforzheim, Germany).

### 2.2. Cell Culture

Neoplastic glioma cells: U87MG (p53^WT^, PTEN^del^, and p14/p16^del^) and T98G (p53^mut^, PTEN^mut^, and p14/p16^del^) human GBM cells were obtained from ATCC, LN-319 (p53^WT^, PTEN^mut^, and p14/p16^WT^) and LN-308 (p53^del^, PTEN^mut^, and p14/p16^WT^) human astrocytoma cells and human LNT-229 (p53^WT^, PTEN^WT^, and p14/p16^del^) GBM cells were kindly provided by M. Hegi (Lausanne, Switzerland). The cell lines were described in detail in [44, 45]. SMA-560 mouse astrocytoma cells were a friendly gift of D. Bigner (Duke University, Durham, NC, USA [46]). Human MZ-18 GBM cells (p53^mut^) were obtained from D. Kögel (Department of Neurosurgery, University Clinics, Frankfurt/Main, Germany [47]). Nonneoplastic cells: immortalized human astrocytic SV-GA cells were obtained from R. Atwood (Brown University, Providence, Rhode Island, USA [48]), primary human brain microvascular pericytes (HBVP) were purchased by ScienCell (San Diego, USA), and immortalized human brain endothelial cells (hCMEC/D3) were obtained from B. Weksler (Weill Cornell Medical College, New York, USA [49]). Glioma and SV-GA cells were maintained in Dulbecco's modified Eagle's medium (DMEM, GIBCO Life Technologies, Eggenstein, Germany) containing 10% fetal calf serum (FCS, GIBCO Life Technologies), penicillin (100 U/mL), and streptomycin (100 *µ*g/mL). hCMEC/D3 cells were maintained in EBM-2 (Lonza, Basel, Switzerland) containing EGM-2 growth factors (Lonza, Basel, Switzerland) in rat tail collagen I (Enzo Life Sciences, New York, USA) coated flasks. HBVP cells were maintained in pericyte basal medium (ScienCell, San Diego, USA) supplemented with 2% inactivated FCS, 1% of pericyte growth supplement (ScienCell, San Diego, USA), penicillin (100 U/ml), and streptomycin (100 *µ*g/ml) in Poly-L-Lysine (Sigma-Aldrich, Taufkirchen, Germany) coated flasks. The cell lines were routinely tested to be free of mycoplasma using the MycoAlert™ Mycoplasma Detection Kit (Lonza GmbH, Cologne, Germany).

### 2.3. Propidium Iodide Staining of Murine Hippocampal Slice Cultures

Murine hippocampal organotypic slice cultures were prepared as previously described [50]. After one week in culture the slices were treated for 24 or 48 h with ISCADOR Qu, Aviscumine, and native ML-1 or were left untreated. Propidium iodide (PI, 5 mg/ml, Sigma-Aldrich, Taufkirchen, Germany) was added in a concentration of 1:2000 to the medium. After incubation for 1 h at 37°C, four pictures were taken each and PI positive cells were counted manually. As a positive control for the induction of cell death the slices were treated for 30 min with 10 *µ*M N-methyl-D-aspartate (NMDA) 24 h in advance to the PI staining.

### 2.4. Cell Growth, Cytotoxicity, Clonogenic Survival, and Cell Cycle Analysis

To determine cell growth, the cells were seeded at 1000 cells/well (growth curve) or 10.000 cells/well (cytotoxicity) in microtiter plates, were allowed to attach overnight, and were then treated with increasing concentrations of the drugs. Cell density was measured 24 and 48 h after treatment by crystal violet staining as previously described [51]. To measure clonogenic survival, LNT-229 (500 cells/well) or LN-308 cells (2500 cells/well) were seeded in 6-well plates and allowed to attach. Cells were treated with either TMZ and/or ML-containing drugs for 24 h and/or were irradiated 24 h after treatment using a Gammacell GC40 irradiator (Nordion UK, Abingdon, Oxfordshire, UK). After treatment, the medium was changed to standard growth medium. After approximately 10 doubling times visible colonies (> 50 cells) were stained with crystal violet and counted manually. To measure clonogenic survival in SMA-560 cells, 500 cells/well were seeded in 96-well plates and treated as above. The assay was stopped when control-treated cells grew to confluency. Cell density was measured by crystal violet staining as previously described [51]. Synergistic and additive effects were calculated using the method of Webb [52]. For cell cycle analysis the cells were treated with ISCADOR Qu, Aviscumine, and native ML-1 for 24 or 48 h or were left untreated. Cell cycle distribution was measured by 5-bromo-2-deoxyuridine (BrdU) incorporation (10 *µ*M, 30 min) followed by addition of a FITC-coupled anti-BrdU antibody (BioLegend, Fell, Germany) and PI as described [53]. Analyses were performed using a Cyan ADP flow cytometer (Beckman Coulter GmbH, Krefeld, Germany) and FlowJo software (FlowJo LLC, Ashland, USA). Blocking of ML activity was performed by adding a polyclonal rabbit pan-anti-ML antibody (ISCADOR AG) to the drug-containing medium 30 min before treatment start.

### 2.5. Quantitative RT-qPCR

For quantitative reverse transcription polymerase chain reaction (RT-qPCR), RNA of treated and untreated cells was isolated using the RNApure isolation kit (Machery-Nagel, Düren, Germany) and transcribed into cDNA by Superscript II (Invitrogen, Karlsruhe, Germany). RT-qPCR was performed using SYBR green master mix (Thermo Fisher Scientific, MA, USA) on an ABI7500 system. Cycling conditions were as follows: 95°C for 10 min, 45 cycles at 95°C for 15 s, 60°C for 1 min, and 72°C for 20 s. Relative mRNA expression was quantified ([EΔCT (gene of interest)/EΔCT (housekeeping gene)]). The following primers were used: GAPDH forward 5′-TGCACCACCAACTGCTTAGC-3′; GAPDH reverse 5′- GGCATGGACTGTGGTCATGAG-3′; CCND1 forward 5′-TGAGGGACGCTTTGTCTGTC-3′; CCND1 reverse 5′-GCCTTTGGCCTCTCGATACA-3′; CCND2 forward 5′- CTGGGTGCTGTCTGCATGTT-3′; CCND2 reverse 5′-AGGTTCCACTTCAACTTCCCC-3′; CCND3 forward 5′-CCGAAACTTGGCTGAGCAGA-3′; CCND3 reverse 5′-GTGTTTACAAAGTCCGCGCC-3′; HDAC6 forward 5′-AAGGTCGCCAGAAACTTGGT-3′; HDAC6 reverse 5′-TGGGGGTTCTGCCTACTTCT; PPP2CA forward 5′- TGGTGGTCTCTCGCCATCTA-3′; PPP2CA reverse 5′- TGACCACAGCAAGTCACACA-3′; ATM forward 5′-CCGAATGTTTTGGGGCAGTG-3′; ATM reverse 5′- TTTTCTCCGTTAGCCACGCA-3′.

### 2.6. Immunofluorescence

5x10^4^ glioma cells were seeded on poly-L-lysine coated glass cover slips and were fixed with 4% paraformaldehyde. After washing with phosphate buffered saline (PBS) and blocking (10% goat serum, 0.3% Triton X-100, PBS), the cover slips were incubated overnight with a CD75s specific antibody (clone LN-1, sc-6263, Santa Cruz, TX, USA) at 4°C. For visualization, a Texas Red coupled secondary antibody (sc-2983, Santa Cruz, TX, USA) was added for 1 h. The cover slips were mounted with VECTASHIELD/DAPI Hard Set mounting medium (VECTOR, Burlingame, CA, USA). Human brain and GBM cryosections of seven different patients were kindly provided by the biobank of the Institute for Pathology (University of Tübingen, ethical approval 475/2016BO2). The human samples as well as cryosections prepared from mouse brain and spleen were fixed, stained, and analyzed for CD75s expression as described above. A FITC-labeled secondary antibody (sc-2082, Santa Cruz, TX, USA) was used. CD75s staining was examined using a Zeiss Imager.Z1 microscope (Carl Zeiss, Oberkochen, Germany). To quantify CD75s expression, at least four pictures per condition were taken and CD75s staining intensity was quantified using ImageJ.

### 2.7. Animal Experiments

For induction of glioma, 75000 human LNT-229 cells were implanted in the right striatum of 5-6-week old female Rj:NMRI Foxn1^nu^/Foxn1^nu^ nude mice (Janvier, St. Berthevin, France) using a mouse stereotactic device with automated infusion pump (Stoelting, Wood Dale, Illinois, USA) and a 10 *µ*l Hamilton syringe (Type 701SN, Medchrom, Flörsheim am Main, Germany) at a rate of 1 *µ*l per minute. At day 10 after tumor cell implantation the mice were randomly split into 8 groups (n=6-8 animals per group, a total of 60 mice). A single dose of Aviscumine (3 *µ*l, 240 ng/ml) was injected intratumorally. As a control some groups obtained an intratumoral injection of PBS. At days 10, 18, and 25 after tumor cell implantation mice were intraperitoneally (i.p.) injected with TMZ (1.5 mg/kg) or PBS as previously described [54] and/or were tumor-irradiated with 3 Gy (6 MV photons) using a LINAC 6C linear accelerator at day 11 after tumor cell implantation as described [55]. The mice were euthanized by lethal CO_2_ inhalation at the onset of tumor-related symptoms. No body weight loss was observed at this time point. All animal experiments were performed according to the German law, Guide for the Care and Use of Laboratory Animals (approval N6/16 of the regional council Tübingen, approval FK/K5112 of the regional council Frankfurt/Main).

### 2.8. Statistical Analysis

The figures show the mean or one representative experiment of at least three independent experiments as indicated ± standard deviation (SD). Quantitative* in vitro* data was assessed for significance by unpaired Student's t-test (*∗p *< 0.05; *∗∗p *< 0.01; *∗∗∗p *< 0.001) using Excel (Redmond, WA, USA). Mouse survival and the median survival time were analyzed with the Kaplan-Meier estimate and tested for significance by the log-rank test (*∗p *< 0.05; *∗∗p *< 0.01; *∗∗∗p *< 0.001) using JMP 13 software (SAS, Böblingen, Germany).

## 3. Results

### 3.1. Cell Death Induction by ISCADOR Qu, Aviscumine, and Native ML-1 in Glioma Cells, Nonneoplastic Cells, and Murine Hippocampal Slice Cultures

**Figure 1:**
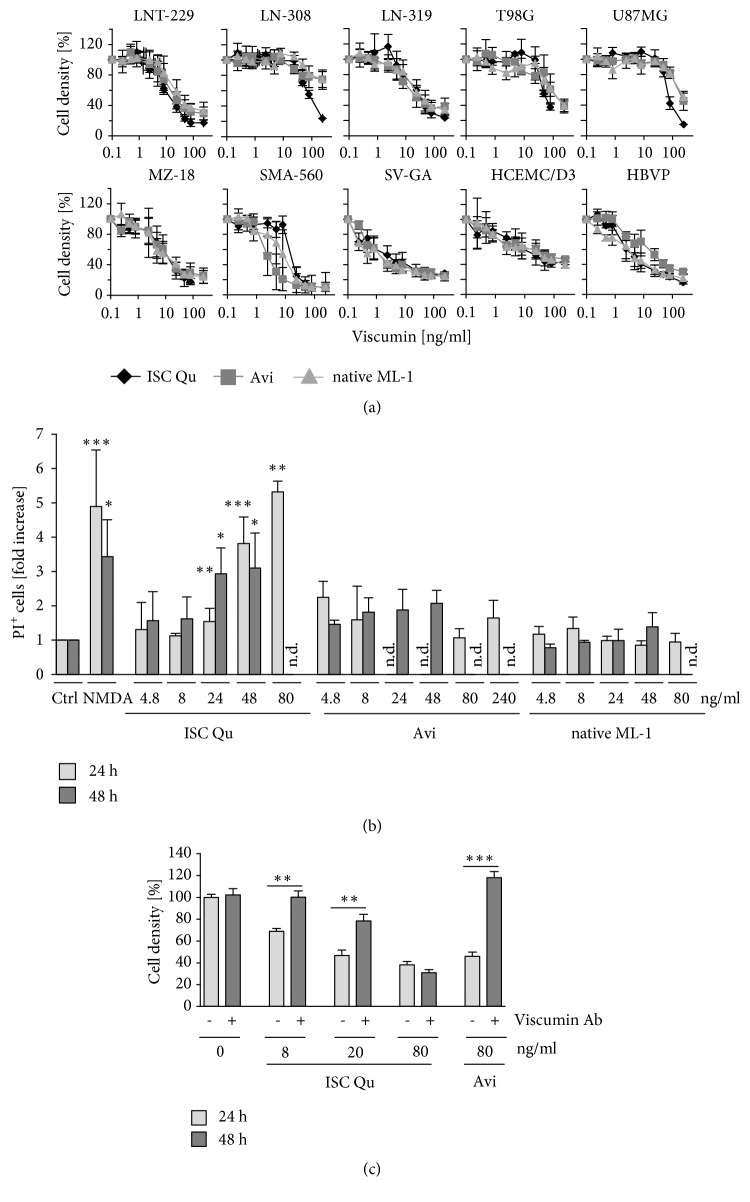
*Induction of cell death by ML-containing drugs*. (a) Cells were treated with increasing concentrations of ISCADOR Qu (ISC Qu), Aviscumine (Avi), or native ML-1 for 48 h and cell density was determined by crystal violet staining. (b) Mouse hippocampal brain slice cultures were treated for 24 h (light gray) or 48 h (dark gray) or with NMDA as a positive control of cell death induction. Dead cells were counted (n=4, SD; nd: not determined). (c) LNT-229 cells were treated with ISCADOR Qu or Aviscumine in the absence or presence of a ML-neutralizing antibody (10 *µ*g/ml) and cell density was measured 48 h later by crystal violet staining (n=3; ±SD; Student's t-test *∗*P < 0.05, *∗∗*P < 0.01, and *∗∗∗*P < 0.001; n.d.: not determined).

We have previously published IC_50_-values for the treatment of LNT-229 GBM cells using ISCADOR Qu, Aviscumine, or native ML-1 [15, 56]}. To evaluate cell death-inducing effects of viscumins in glioma cells in general, we included additional human (LN-308, T98G, LN-319, U87MG, and MZ-18) and mouse glioma (SMA-560) as well as human nonneoplastic cell lines (SV-GA, hCMEC/D3) and primary cells (HBVP) in this study. Aviscumine and native ML-1 were less toxic compared to ISCADOR Qu. In most cells Aviscumine and native ML-1 induced cell death 48 h after treatment in a dose dependent manner (Figure 1(a)), whilst this effect was already detectable 24 h after treatment with ISCADOR Qu [12]. To determine sensitivity of mammalian cells toward ML-induced cell death, we calculated IC_50_ values for nonneoplastic and neoplastic cells (Table 1). In general, glioma cells differ in their sensitivity towards ML. LN-308, T98G, and U-87MG glioma cells were only moderately sensitive whilst LNT-229, LN-319, MZ-18, and murine SMA-560 glioma cells were sensitive. We additionally performed a correlation analysis to identify whether known mutations in the human glioma cell lines correlate to ML-sensitivity, but no correlation in the context of p53, PTEN, or p14/p16 mutations has been identified. All nonneoplastic cells tested so far showed cell death induction after the treatment and IC_50_-values at 48 h were in the range of those observed for sensitive glioma cells.

To determine toxic effects of ISCADOR Qu, Aviscumine, and native ML-1 in nonneoplastic brain tissue* ex vivo*, we used murine hippocampal slice cultures and determined the induction of cell death after addition of the drugs. Even using high concentrations of Aviscumine or native ML-1, very low or even no cell death induction in hippocampal mouse brain tissue was observed up to 48 h after treatment. In contrast, ISCADOR Qu induced cell death much faster and at a concentration of > 24 ng/ml of ML compared to Aviscumine and native ML-1 (Figure 1(b)). Since ISCADOR Qu contains, besides ML, also VT and minor compounds like triterpenes, flavonoids, phytosterols, and oligo- and polysaccharides that might influence the efficiency or that additionally induce cell death, we added a pan-ML-neutralizing antibody and analyzed cell death induction in treatment-sensitive human LNT-229 glioma cells. As demonstrated in Figure 1(c), in human LNT-229 glioma cells Aviscumine-induced cell death after 48 h incubation was completely abolished by adding the ML-antibody, even at an Aviscumine concentration of 80 ng/ml that is much higher than the IC_50_ of 17.7 ng/ml we determined in cell culture. In contrast, cell death induced by ISCADOR Qu was only abolished by the ML-antibody at a low concentration counting for 8 ng/ml of ML and was partially reduced at a concentration counting for 20 ng/ml of ML. The protective effect of the ML-antibody was completely gone at a concentration of 80 ng/ml of ML in the VE ISCADOR Qu, indicating that at this concentration cell death induction was ML-independent and that the elevated cytotoxic effect of ISCADOR Qu at higher concentrations we observed in brain slice cultures and in human and mouse cells was mainly a result of VTs and of the minor compounds that are additionally present in the extract.

### 3.2. Expression of CD75s on Glioma Cells and Tissue

**Figure 2:**
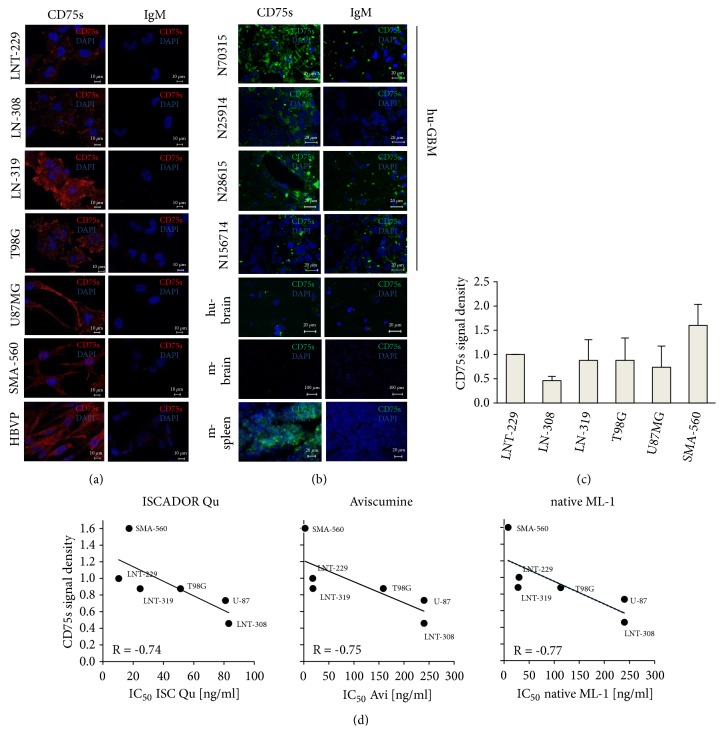
*CD75s expression in GBM cells and tissue*. (a/b) CD75s staining in glioma cell lines and HBVPs (a), in human and murine tissue, and in human glioma specimen (b). Representative pictures of three independent experiments are shown. (c) Relative CD75s signal density (n=3; SD). (d) Correlation analysis of the CD75s expression and IC_50_ values (48h) of viscumin treatment (R, relation coefficient).

It has been published that the MLs bind to CD75s on the cell surface of target cells which enables its uptake into the cell [26, 27]. To determine CD75s expression in glioma and to analyze whether CD75s expression correlates to cell death induced by the ML-containing drugs, we quantified CD75s expression in the glioma cell lines (LNT-229, U87MG, LN308, LN-319, and SMA-560) by immunofluorescence microscopy. All human and mouse glioma cell lines expressed CD75s, but to a different extent (Figures 2(a) and 2(c)). We also analyzed CD75s expression in nonneoplastic human primary pericytes (HBVP) since we found these cells also to be treatment-sensitive (Figure 1(a), Table 1). HBVPs expressed high amounts of CD75s (Figure 2(a)) which correlates well with their treatment-sensitivity.

In contrast to normal human or mouse brain where we could not detect CD75s expression, in GBM tissue CD75s expression varied. 5/7 GBM were positive, 1/7 showed slight expression, and 1/7 was negative (exemplarily shown in Figure 2(b) and Table 2). As a positive staining for CD75s we used mouse spleen since it has been published that CD75s is highly expressed on immune cells [30]. In GBM we did not observe any correlation of CD75s expression to IDH1- or MGMT-mutations. However, there was a clear correlation of CD75s expression and ML-vulnerability, at least in the human and murine glioma cell lines (Figure 2(d)). This suggests that CD75 expression on target cells might define their treatment-sensitivity and that CD75s might be a GBM associated marker that can be used to estimate the efficiency of ML-drug-based therapy in glioma.

### 3.3. ISCADOR Qu, Aviscumine, and Native ML-1 Reduce Growth Rate and Modify Cell Cycle Distribution in Glioma Cells

**Figure 3:**
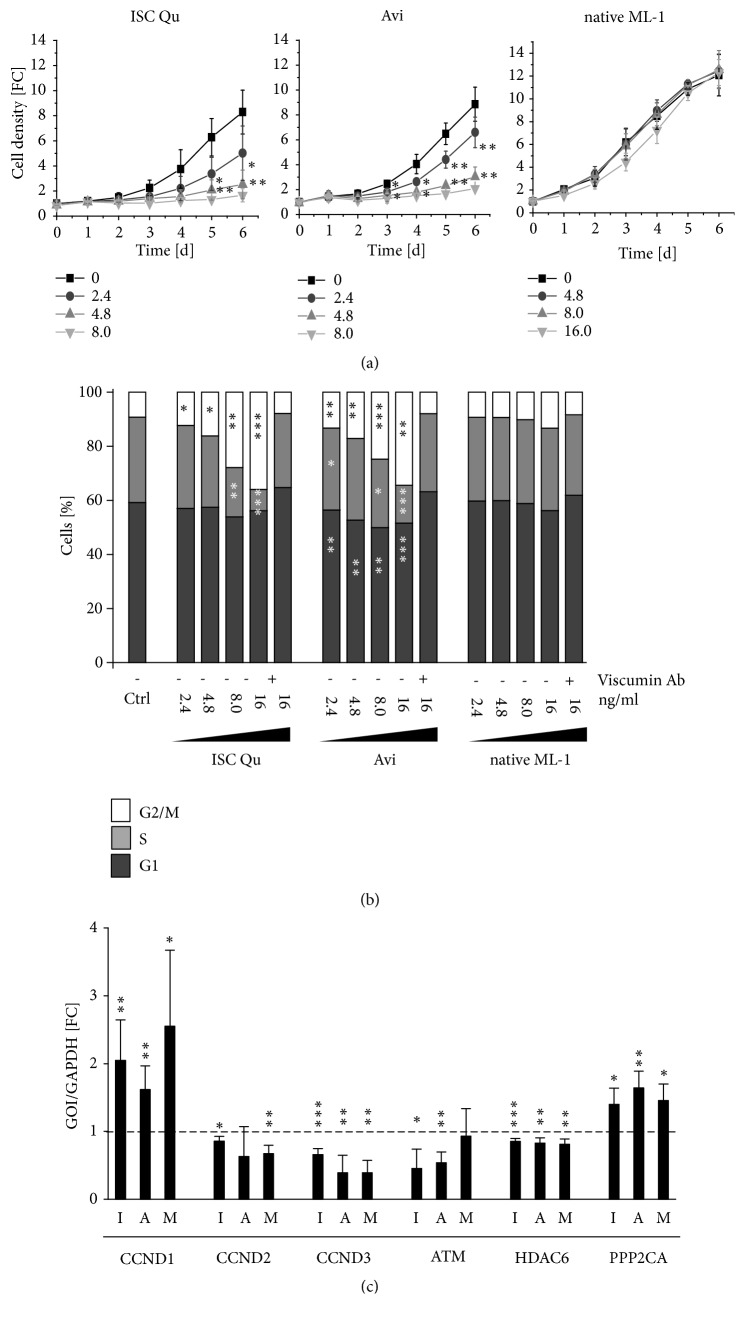
*Drug-induced modulation of proliferation and cell cycle distribution in glioma cells*. (a) Growth curve of ML-treated LNT-229 glioma cells. Cells were treated for 24 h with ISCADOR Qu (ISC Qu), Aviscumine (Avi) (0, 2.4, 4.8, or 8 ng/ml), or native ML-1 (0, 4.8, 8, or 16 ng/ml). After treatment the medium was changed to drug-free growth medium and cell growth was measured by crystal violet staining every 24 h (FC, fold change). (b) Cell cycle distribution of treated LNT-229 glioma cells 48 h after treatment. The cells were treated with ISC Qu, Avi, or native ML-1 at increasing concentrations in the presence of absence of a ML-specific antibody (4.8 *µ*g/ml). (c) RT-qPCR analysis of LNT-229 cells treated for 24 h with 8 ng/ml ISCADOR Qu (I), Aviscumine (A), or native ML-1 (M). The dashed line demonstrates baseline expression in untreated control cells (GOI gene of interest; FC fold change; n=3; ±SD; Student's t-test *∗*P < 0.05, *∗∗*P < 0.01, and *∗∗∗*P < 0.001).

VE and ML are known to reduce cancer cell growth both* in vitro* and* in vivo* [12, 17, 24, 56–59]. Besides induction of cell death, reduction in tumor cell growth could also be an effect of reduced proliferation. We evaluated whether this is the case also for glioma and tested several glioma cells lines (Figure 1 and data not shown). To this end, we treated the cells with ISCADOR Qu, Aviscumine, or native ML-1 at increasing concentrations for 24 h followed by incubation in ML-free growth medium and measured cell density every 24 h. As shown exemplarily for human LNT-229 GBM cells, both ISCADOR Qu and Aviscumine were potent inhibitors of cell growth even at low concentrations, whereas native ML-1 did not reduce cell growth at any concentration tested so far (Figure 3(a)). We further analyzed cell cycle distribution. Even at a concentration of 2.4 ng/ml ML which is far below the IC_50_ determined for LNT-229 cells, Aviscumine and ISCADOR Qu decreased the cell counts in the S-phase and induced an accumulation of cells in G_2_/M. Higher concentrations of Aviscumine or ISCADOR Qu further increased the population of arrested G_2_/M cells. Interestingly, but in accordance to its missing function in blocking cell growth, native ML-1 treatment did not change cell cycle distribution at any concentration. The induction of cell cycle arrest was dependent on the functional presence of ML since the addition of a ML-neutralizing antibody completely annulled the proliferation inhibitory activity (Figure 3(b)).

To identify the underlying mechanisms, we analyzed the expression of several genes that regulate the cell cycle using RT-qPCR. In the panel of the cyclins A1, A2, B1, B2, B3, D1, D2, D3, E1, and E2, only the cyclins D1, D2, and D3 were differentially expressed between mock- and ML-treated LNT-229 GBM cells. Cyclin D1 was upregulated whilst cyclins D2 and D3 were downregulated after ML-treatment (Figure 3(c)). We also tested the expression of the cyclin dependent kinase inhibitors CDKN1A (p21) and CDKN1B (p27), but no changes were detectable (data not shown). Since it has been published that histone deacetylases (HDACs) control the functions of key cell cycle proteins and can modulate the G_2_/M phase transition [60], we also analyzed HDAC expression. In the panel of HDACs, HDAC6 was downregulated in LNT-229 GBM cells after treatment (Figure 3(c)). Additionally, expression of ataxia-telangiectasia mutated (ATM) was reduced and the tumor suppressor protein phosphatase 2 catalytic subunit alpha (PPP2CA) mRNA was upregulated (Figure 3(c)). Notably, also native ML-1 led to changes in the expression of cell cycle regulating genes even if no effect of native ML-1 on cell growth and cell cycle distribution was observed. In summary, there is evidence that ML-containing drugs, although to a different extent, reduce the proliferation of glioma cells by inducing cell cycle arrest in the G_2_/M phase of the cell cycle.

### 3.4. Synergistic and Additive Effects of Adjuvant Viscumin Treatment* In Vitro*

**Figure 4:**
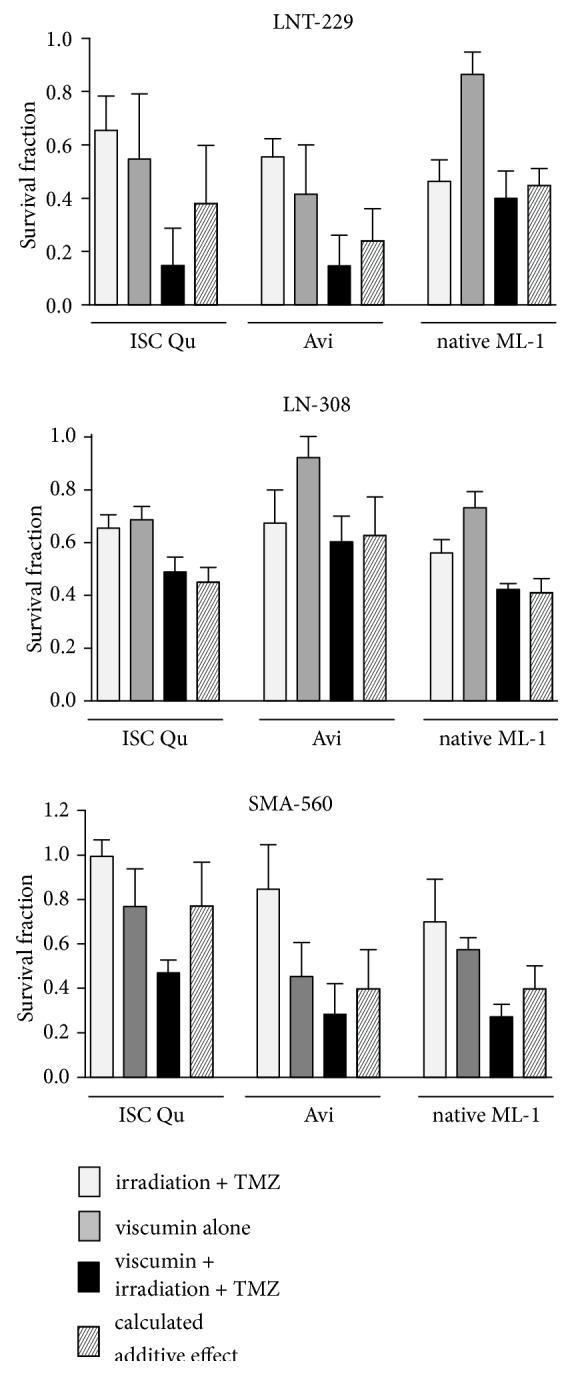
*Additive and synergistic effects*. LNT-229 (upper panel), LN-308 (middle panel), and SMA-560 (lower panel) glioma cells either were exposed to radio-chemotherapy alone (white bar), with viscumin (grey bar), or were exposed to radio-chemotherapy in combination with ISCADOR Qu (ISC Qu), Aviscumine (Avi), or native ML-1 (black bars). The dashed bars indicate the predicted effect of combined treatment according to the fractional product method (for details, see Section 2). The following conditions were chosen: LNT-229: 3 Gy, 3 *µ*M TMZ, 3.6 ng/ml ISCADOR Qu, or Aviscumine, 4.8 ng/ml native ML-1; LN-308: 1.5 Gy, 10 *µ*M TMZ, 16 ng/ml ISCADOR Qu, Aviscumine, or native ML-1; SMA-560: 3 Gy, 30 *µ*M TMZ, 6.5 ng/ml ISCADOR Qu, 1.5 ng/ml Aviscumine, or 2 ng/ml native ML-1 (n=3; ±SD).

Adjuvant treatment of GBM patients with (natural) compounds that work in synergy with glioma standard therapy or even with novel therapeutic approaches might be helpful to optimize therapeutic regimen. To test whether ML-containing drugs enhance the effect of irradiation and TMZ based chemotherapy, we performed colony formation assays using human ML-/TMZ-sensitive LNT-229, human ML-moderately-sensitive/TMZ-resistant LN-308, and murine ML-sensitive/TMZ-resistant SMA-560 glioma cells. We performed standard treatment (irradiation plus TMZ) using concentrations of TMZ that are below the concentration normally measured in the serum of TMZ-treated GBM patients and that are far below the IC_50_ determined for the cell lines used [61, 62], in combination with irradiation. Either alone or in combination with radio-chemotherapy, the cells were treated with ISCADOR Qu, Aviscumine, or native ML-1 at concentrations that are below the IC_50_ values we determined (for the concentrations used in this assay proceed to Figure 4). Clonogenic survival was determined and predicted additive effects were calculated. As shown in Figure 4, adjuvant treatment induced at least additive effects in the reduction of clonogenic survival in all three tested cell lines. Even reaching not a significance level, in ML-sensitive LNT-229 and SMA-560 cells, combined treatment provides more than additive effects than ML alone or TMZ plus irradiation as treatment options. This suggests that the ML-treatment confers, by suppressing the clonogenic survival of glioma cells, beneficial effects if used in combination with radio-chemotherapy.

### 3.5. Effects of Adjuvant Viscumin Treatment* In Viv*o

**Figure 5:**
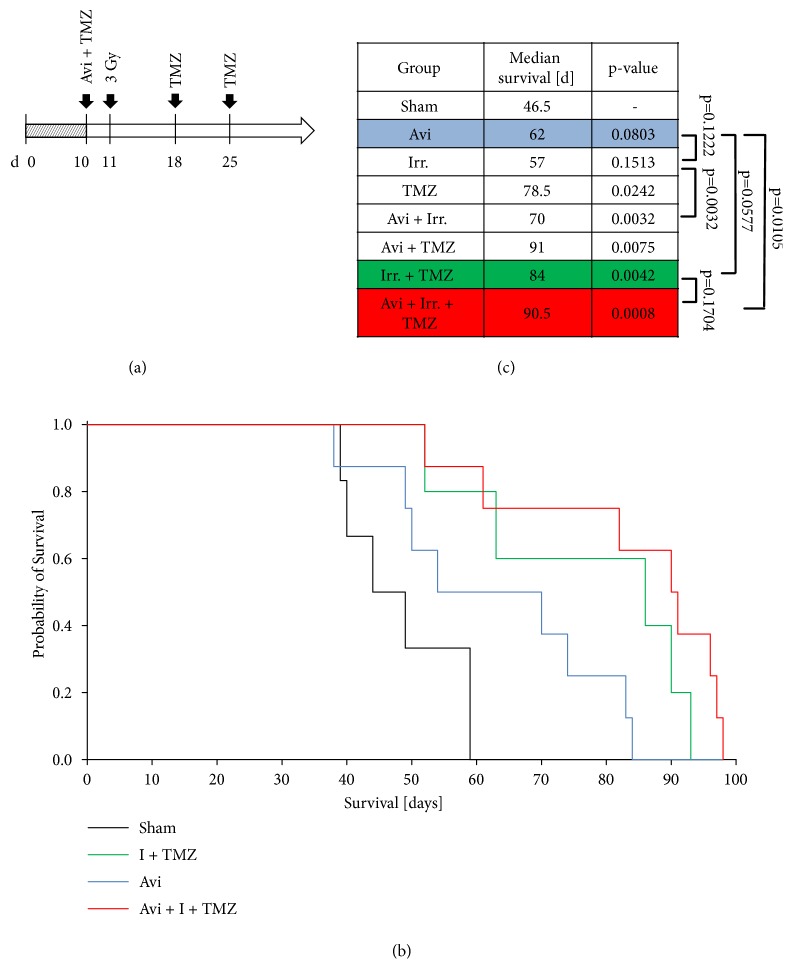
*Adjuvant Aviscumine treatment of glioma bearing mice*. (a) Scheme of the treatment of mice bearing orthotopic LNT-229 tumors. (b) Kaplan Mayer survival curves. Mice were intratumorally sham-injected with PBS or with Aviscumine (Avi, 0.72 ng ML in total, dotted line) at day 10. Focal tumor irradiation (3 Gy) was performed at day 11 followed by weekly intraperitoneal TMZ injections (1.5 mg/kg) for three weeks. Adjuvant Aviscumine treatment resulted in a trend for prolonged survival compared to the radio-chemotherapeutic treatment group and in a significant prolonged survival compared to the control group. (c) Median survival (n=6-8 animals, P-values, Log-rank test).

We also evaluated the effects of the adjuvant treatment* in vivo. *To prove toxicity* in vivo *before using the drugs in a therapeutic mouse model, we injected Aviscumine intracerebrally into NMRI mice in a concentration of up to 720 ng (i.e., 3 *µ*l of 240 ng/m) but observed no behavioral changes or pathological modifications around the injection side at early and late time points after the injection (data not shown). We did not choose ISCADOR Qu in our therapeutic regimen due to the elevated toxicity we had observed in mouse brain tissue* ex vivo *(Figure 1(b)). Furthermore, we did not use native ML-1* in vivo* due to its lower therapeutic efficacy we observed* in vitro*. To determine the effects of Aviscumine in combination with radio-chemotherapy, we used nude mice bearing orthotopically growing LNT-229 GBM. Ten days after tumor cell implantation the mice received a single intratumoral injection of PBS or Aviscumine (720 ng). Radio-chemotherapy was done by focused tumor irradiation (3 Gy) at day 11 [55]; TMZ was applied by weekly intraperitoneal injections (1.5 mg/kg) starting at day 10 (Figure 5(a)). Even if not quite significant, treatment of the mice using Aviscumine monotherapy prolonged the median survival compared to control treated mice (62 d in the Aviscumine group versus 46.5 d in the control group, p=0.0803) and was even superior to tumor irradiation (62 d in the Aviscumine group versus 57 d in the irradiated group, p=0.1222). As seen* in vitro* in cell culture, the combined treatment using Aviscumine plus tumor irradiation was also superior to the single treatments. The median survival time of Aviscumine plus irradiation treated mice was prolonged by 13 days compared to the irradiation treatment alone (57 d in the irradiated group versus 70 d in the irradiation + Aviscumine group, p=0.0032) and by 8 days compared to the Aviscumine treatment (62 d in the Aviscumine group versus 70 d in the irradiation + Aviscumine group, p=0.6892). The overall survival was 65 days in the irradiation group and 89 days in the Aviscumine plus irradiation treatment group (data not shown). Since LNT-229 tumors are highly sensitive to TMZ [62], TMZ alone or in combination with irradiation was very efficient and reaches a prolongation of median survival of 32 days (46.5 d in control mice versus 78.5 d in the TMZ group (p=0.0042) and 84 d in the TMZ + irradiation group (p=0.0042)). Nevertheless, we observed a trend to an additional therapeutic effect if the mice received a single intratumoral injection of Aviscumine. Combined treatment of the mice with Aviscumine plus TMZ prolonged the median survival by 12.5 days compared to TMZ alone (78.5 d in the TMZ group versus 91 d in the Aviscumine + TMZ group, p=0.944). However, overall survival was nearly equal in these groups (data not shown). Aviscumine in combination with TMZ + irradiation further prolonged the median survival by 6.5 d (90.5 d in the combined treatment group versus 84 d in the TMZ + irradiation group, p=0.1704; Figures 5(b) and 5(c)) suggesting that an adjuvant Aviscumine treatment conveys a positive effect if used in combination with an irradiation based monotherapy or with the standard GBM treatment.

## 4. Discussion

Therapeutic regimen for GBM standardly includes optimal surgical resection of the tumor, followed by tumor irradiation and TMZ-based chemotherapy. However, even under optimal conditions, recurrence is inevitable. The median survival of GBM patients is only 15 months using the standard “Stupp” protocol [63] that includes TMZ-based radio-chemotherapy. This period could be further extended to nearly 21 months if patients receive a tumor treating field therapy [1]. With the background of this infaust prognosis, several novel therapeutic strategies have been developed and have been tested in preclinical and clinical studies. These novel strategies either target tumor angiogenesis, inhibit receptor tyrosine kinases, mitigate tumor-associated immunosuppression, push the patient's antitumor immune response, or starve the tumor using ketogenic diet. However, no sweeping achievement in the prolongation of the survival of GBM patients has been accomplished by these novel treatment options until today. In this context, a strategy that targets optimizing the effects of radio-chemotherapy might provide benefit for GBM patients. In this regard, the identification and application of (natural) compounds that potentiate and optimize the effects of TMZ and irradiation on GBM growth might improve the prognosis and might extent the progression free or overall survival of GBM patients. Extracts of the European mistletoe* Viscum album L*. have been used for decades in the adjuvant treatment of several cancer entities. Antitumor effects of the mistletoe have been demonstrated* in vitro* and* in vivo* using plant extracts, purified native, or recombinant ML-1 [9, 12, 14, 15, 20–24, 40, 56, 64–66]. For GBM, two studies suggested that a complementary treatment with ML-containing drugs in addition to tumor irradiation provides additional therapeutic impact [67, 68]. However, little is known if and to which extent ML-containing drugs are feasible agents to optimize GBM standard therapy and which are the mechanisms by which they provide these effects.

In this regard we analysed the effects of ISCADOR Qu, a ML-rich VE, Aviscumine, a recombinant ML-1, and native ML-1, isolated from mistletoe plants growing on ash trees, on glioma cell growth, proliferation, clonogenic survival, and their capability of working in synergy with tumor irradiation and TMZ-based chemotherapy. Additionally, we measured toxic effects of these drugs in nonneoplastic cells and in normal brain tissue. For glioma, we tested a panel of 6 human and one mouse glioma cell lines with different genetic background towards their vulnerability to the treatment. 3/7 glioma cell lines (LN-308, T98G, and U87MG) were only moderately sensitive with an IC_50_ (48 h) > 50 ng/ml of ML in ISCADOR Qu, > 150 ng/ml of Aviscumine, and >100 ng/ml of native ML-1 whilst 4/7 glioma cells were sensitive (LNT-229, LN-319, MZ-18, and SMA-560). There was no correlation between the p53, PTEN, or p14/16 mutational status and the drug-sensitivity in the panel of glioma cells we tested so far. Drug-sensitivity correlated well to the expression of the ML receptor CD75s on the glioma cells (Figure 2(d)). There might be further parameters like differences in drug sensitivity or cell motility that might influence the glioma cell's vulnerability towards ML-based drugs. To clarify this, further investigation will be necessary.

In nonneoplastic cells of the brain like endothelial (HCMEC/D3), astrocytic (SV-GA), or pericytic cells (HBVP), the ML-based preparations we tested also reduced proliferation and induced cell death in the same range as in GBM cells (Table 1). Being very important with regard to a ML-based therapy in patients, the toxicity we detected in nonneoplastic cell cultures did not reflect the* in vivo* toxicity in the brain tissue, since Aviscumine and native ML-1 did not induce cell death in organotypic hippocampal brain tissue culture or mouse brains, even at a concentration of 240 ng/ml, a concentration we found to be highly toxic for most cultures of nonneoplastic primary or immortalized cells (Figure 1(b), Table 1). Since we observed CD75s expression in primary HBVPs in culture (Figure 2(a)), but not in pericytes nor in astrocytes of healthy human and mouse brain tissue (Figure 2(b)), the sensitivity of nonneoplastic cultured cells towards the ML-containing drugs might be, among other explanations, due to an artificial upregulation of CD75s expression in these cells under cell culture conditions. In 6/7 human GBM tissue samples, but not in the healthy human brain, CD75s was expressed (Figure 2(b), Table 2). We assume that the sensitivity towards the drug treatment mainly depends on the expression of the ML-receptor CD75s which is expressed in all glioma cells tested so far and in 6/7 GBM tissue, but not in the healthy brain (Figure 2). This fits well with the missing toxicity we have observed for Aviscumine and ML-1 in hippocampal brain tissue* ex vivo* or in mouse brains (Figure 1, data not shown). Besides, in glioma cells we found a correlation between the vulnerability towards ML-induced cell growth inhibition and cell death induction and the expression of CD75s. Our data suggest CD75s to be a putative marker that predicts the therapeutic impact of an adjuvant mistletoe therapy of GBM.

In all cells tested so far ISCADOR Qu was generally more toxic than Aviscumine or ML-1 (Table 1). Additionally, in contrast to Aviscumine and native ML-1, ISCADOR Qu induced cell death in mouse hippocampal slices* ex vivo* (Figure 1(b)). Since Aviscumine, but not ISCADOR Qu induced cell death, was completely abolished by addition of a pan-ML-antibody (Figure 1(c)) we suggest that the enhanced toxicity we have observed for ISCADOR Qu in healthy brain tissue* ex vivo* is mainly an effect of VTs and/or other minor compounds like triterpenes or flavonoids [69–71] that are present in the extract, but not in the recombinant protein Aviscumine, nor in native ML-1.

MLs have been described to inhibit proliferation of tumor cells [18, 58, 72, 73]. Even at a low concentration that is far below the IC_50_ we determined, ISCADOR Qu and Aviscumine reduced cell growth, leading to a reduced amount of S-phase cells and an increase of cells that are arrested in the G_2_/M phase of the cell cycle. These data are in concordance with the results of Weissenstein et al. who showed that* Viscum album* preparations induce G_2_/M arrest in breast cancer cells [9]. Alterations in the cell cycle distribution were accompanied by changes in the expression of cell cycle regulating genes. Cyclin D1 was significantly upregulated whilst cyclins D2 and D3 were slightly downregulated by all three ML preparations (Figure 3(c)). The upregulation of cyclin D1 might explain the fact that the population of G_1_ phase cells slightly decreased upon ISCDAOR Qu and Aviscumine treatment. We found the tumor suppressor PPP2CA to be upregulated by ML. PPP2CA is a negative regulator of the cell cycle and dephosphorylates a variety of cell cycle regulating proteins [74, 75]. Additionally, we found HDAC6 mRNA to be downregulated after ML treatment. HDACs alter acetylation not only of histones, but also of several other proteins like tubulins, and inhibition of HDACs can in turn lead to an arrest in G_2_/M and ultimately to mitotic catastrophe [60, 76–78]. This might explain the arrest of ML-treated LNT-299 glioma cells in G_2_/M. However, it could not explain the missing effects of native ML-1 treatment on cell cycle distribution since cell cycle regulating genes were also differentially regulated by native ML-1. Until today it is also not clarified in detail by which mechanism MLs regulate gene transcription. This might be a direct effect of ML on the transcriptional machinery or an indirect effect by their RIP-function leading to the translational inhibition of a variety of proteins involved in transcriptional or posttranscriptional processes. Finally, the mechanisms how VE or MLs modulate cell cycle distribution and induce an arrest in the G_2_/M phase of the cell cycle remain obscure.

ML-containing drugs are not intended as a single agent treatment for GBM patients, but as complementary therapy in addition to the current GBM treatment using irradiation and TMZ based chemotherapy. Performing adjuvant treatment experiments in cell culture, we found at least additive cytostatic effects by treating the cells with Aviscumine, ISCADOR Qu, or native ML-1 in combination with radio-chemotherapy. This combination reached more than additive effects in ML-sensitive human LNT-229 and murine SMA-560 glioma cells (Figure 4). The additive effects might be explained by the differential expression of genes like PPP2CA, ATM, and HDAC6 in the ML-containing drug treated cells. Particularly ATM, PPP2CA, and HDACs are known to mediate the vulnerability of tumor cells towards irradiation and chemotherapeutic drugs. In this context it has been published that ATM is upregulated in glioma and its inhibition can reduce glioma growth and sensitizes glioma cells towards ionizing radiation and TMZ [79–83]. Secondly, PPP2CA is known to interact with ATM [84]. Thirdly, HDAC6 inhibition can enhance the effect of irradiation and TMZ in GBM [85, 86]. Lastly, cyclin D1 overexpression, as induced by the three substances, reportedly induces replication-associated DNA double-strand breaks (DSB) due to forced G_1_ to S transition. If ATM is additionally downregulated, the DSB repair machinery is inhibited, and cell death increased in tumor cells [87].

Due to the toxic effects of VTs or other minor components that are present in ISCADOR Qu (Figure 1(b)) and due to low efficacy of native ML-1 treatment* in vitro* (Figure 3(a)), we performed* in vivo* experiments only with Aviscumine. Despite being not significant, there is a trend that a single intratumoral injection of Aviscumine provides therapeutic impact since it prolonged the survival of GBM bearing mice for 15.5 days and seemed to be even better than tumor irradiation alone (Figure 5(c)). Since we used TMZ sensitive LNT-229 cells in our mouse model, TMZ-based chemotherapy alone or in combination with irradiation significantly prolonged the survival of tumor bearing mice. Importantly, adjuvant Aviscumine therapy, especially in combination with irradiation, prolonged the survival of the mice and further prolonged the survival if used in combination with TMZ plus irradiation (Figure 5). In our approach we used a single intratumoral injection of Aviscumine, so one could speculate that repeated intratumoral Aviscumine injections might further enhance its therapeutic effect. The intratumoral application of VE is not a common procedure in cancer patients; however, it has been tested to be safe in a variety of peripheral tumors. In the last years this application form came more and more into the focus of clinical physicians to treat solid tumors [88]. To treat GBM, intratumoral drug delivery or delivery of oncolytic viruses is a promising approach to overcome the blood-brain-barrier and to get maximum drug concentrations in the tumor. However, until today it is also not a common treatment in the clinic (for review see [89]). With regard to GBM, an intratumoral injection of VE might be of benefit since by this application the formation of anti-ML antibodies, a well-known effect of the mistletoe therapy, might be suppressed.

It has been described that VE stimulate the immune system [13, 14, 56, 90]. A major limitation using a xenograft mouse glioma model as we did will be the missing immune system in these mice; therefore antitumoral immune responses of the mistletoe therapy cannot be detected by this approach. Additionally, xenograft human GBM in mice did not grow that invasively as it is the situation in GBM patients. So we can only speculate that the beneficial effects we observed by treating glioma bearing mice with Aviscumine alone or in combination with irradiation and/or TMZ will provide benefit or will be even better in GBM patients than in mice. In this regard it has been demonstrated in an immunocompetent rat urinary bladder model that animals that received weekly instillations with Aviscumine showed significantly lower rates of atypical hyperplasia and neoplastic transformation of the bladder tissue [91]. In the treatment of malignant melanoma patients, repeated subcutaneous injections of Aviscumine led to an enhancement of the overall survival [92]. However, if the benefit of the adjuvant intratumoral ML-therapy in mice we observed will reflect the situation in GBM patients in the clinic might be debatable. A broad systematic review regarding the use of VE in solid tumors, which also includes glioma, did not show any benefit in the patient's survival [93]. However, none of the GBM-associated trials described in this review used an intratumoral ML treatment.

## 5. Conclusions

In the treatment of GBM, despite intensive research, no relevant prolongation of survival was achieved in the last decades. Currently, only irradiation plus TMZ-based chemotherapy, combined with tumor treating fields, provides a short, but significant prolongation of the median survival. We have identified mistletoe-based drugs as agents that work in synergy with irradiation or TMZ-based chemotherapy in the treatment of glioma. Using* in vitro* cell culture assays as well as a mouse xenograft GBM model and adjuvant mistletoe treatment, we conclude that an intratumoral mistletoe therapy in addition to irradiation and/or chemotherapy might provide benefit in the treatment of GBM.

## Figures and Tables

**Table 1:** IC_50_-values [ng/ml of ML] for ISCADOR Qu, Aviscumine, and native ML-1 in glioma and nonneoplastic cell lines. Cell density was measured by crystal violet. IC_50_-values were calculated from the mean of at least three independent experiments (del: deleted, mut: mutated, WT: wild type, and n.d.: not determined).

Cell line				IC_50_ 24 h			IC_50_ 48 h		
	p53	PTEN	p14/16	ISC Qu	Avi	ML-1	ISC Qu	Avi	ML-1
LNT-229	WT	WT	del	37.09	95,60	>240	10.50	17.78	29.73
LN-308	del	mut	WT	139.72	>240	>240	83.13	>240	>240
LN-319	WT	mut	WT	71.36	>240	>240	24.64	18.23	27.95
U87MG	WT	del	del	140.60	>240	>240	81.03	>240	>240
T98G	mut	mut	del	101.19	>240	>240	51.24	158.81	113.11
MZ-18	mut	n.d.	n.d.	35.45	>240	>240	11.56	10.94	9.97
SMA-560	n.d.	n.d.	n.d.	35.70	10.23	19.25	17.31	2.69	8.25

SV-GA				65.95	>240	>240	2.36	1.37	1.02
HBVP				28.86	>240	58.37	4.85	23.59	5.01
hCMEC/D3				>240	>240	>240	27.92	105.12	42.02

**Table 2:** Characteristics of human GBM tissues and respective levels of CD75s staining. CD75s staining intensity was compared to IgM control staining and then classified as negative (-), slightly positive (+/-), positive (+), or strongly positive (++).

	Primary/ secondary GBM	IDH^R132H^ mutation	IDH sequence	MGMT status	CD75s
N28615	pGBM	Negative	n.d.	Unmethylated	+
N70315	pGBM	Negative	n.d.	Unmethylated	++
N156714	pGBM	Negative	n.d.	Unmethylated	-
N125014	pGBM	Negative	n.d.	Unmethylated	-/+
N152509	sGBM	Positive	n.d.	Methylated	++
N25914	sGBM	Negative	R132G	Methylated	++
N165014	sGBM	Negative	R132G	Methylated	+
hu brain	- - - - - - -	- - - - - - - -	- - - - - - - -	- - - - - - - - -	-

## Data Availability

All data analyzed during this study either are included in this published article or are available from the corresponding author upon request. Since no large datasets were generated during the current study they are not publicly available but are available from the corresponding author upon reasonable request.

## Abbreviations

Avi: Aviscumine
ATM: Ataxia-telangiectasia mutated
BrdU: 5-Bromo-2-deoxyuridine
CD75s: *α*2-6 Sialo-*N*-acetyllactosamine
CDKN1A: Cyclin dependent kinase inhibitor 1A, p21
CDKN1B: Cyclin dependent kinase inhibitor 1B, p27
CDKN2A: Cyclin dependent kinase inhibitor 2A, p16
FC: Fold change
FCS: Fetal calf serum
GBM: Glioblastoma
GOI: Gene of interest
HBVP: Human brain microvascular pericytes
HDACs: Histone deacetylases
IC_50_: Inhibitory concentration of 50%
ISC Qu: ISCADOR Qu
ML: Mistletoe lectins
ML-1: Mistletoe lectin 1
n.d.: Not determined
NMDA: N-Methyl-D-aspartate
PBS: Phosphate buffered saline
PI: Propidium iodide
PPP2CA: Tumor suppressor protein phosphatase 2 catalytic subunit alpha
PTEN: Phosphatase and Tensin homolog
R: Relation coefficient
RIP: Ribosomal inhibitor type 2 proteins
SD: Standard deviation
TMZ: Temozolomide
VE: *Viscum album L.* extracts
VT: Viscotoxins.
